# Prevalence of *ARID1A* Mutations in Cell-Free Circulating Tumor DNA in a Cohort of 71,301 Patients and Association with Driver Co-Alterations

**DOI:** 10.3390/cancers14174281

**Published:** 2022-09-01

**Authors:** Razelle Kurzrock, Charu Aggarwal, Caroline Weipert, Lesli Kiedrowski, Jonathan Riess, Heinz-Josef Lenz, David Gandara

**Affiliations:** 1 WIN Consortium for Personalized Cancer Therapy, BP 90059, 94801 Villejuif, France; 2 Genomic Sciences and Precision Medicine Center, Medical College of Wisconsin, Milwaukee, WI 53226, USA; 3 Division of Hematology and Oncology, Department of Medicine, University of Pennsylvania Perelman School of Medicine, Philadelphia, PA 19104, USA; 4 Guardant Health, Palo Alto, CA 94304, USA; 5 Division of Hematology and Oncology, University of California Davis Comprehensive Cancer Center, Sacramento, CA 95817, USA; 6 Norris Comprehensive Cancer Center, Keck School of Medicine, University of Southern California, Los Angeles, CA 90033, USA

**Keywords:** *ARID1A*, cancer, liquid biopsy, cfDNA

## Abstract

**Simple Summary:**

*ARID1A* abnormalities disturb gene coding processes and correlate with immunotherapy responsiveness. We report the first blood sample-based genomic sequencing of *ARID1A* in DNA shed from tumors into the circulation (known as cell-free DNA (cfDNA) from liquid biopsy). Altogether, of 62,851 cancer patients with ≥1 cfDNA alteration in the blood, 3137 (5%) had ≥1 deleterious *ARID1A* alteration (a frequency similar to the ~6% generally reported in tissue sequencing), suggesting this non-invasive test’s value in detecting *ARID1A*. *ARID1A* alterations were most frequent in endometrial (21.3% of patients) and bladder cancer (12.9% of patients). As compared to blood samples without *ARID1A* aberrations, those with a functional (deleterious) *ARID1A* abnormality had more DNA alterations/sample (median, 6 versus 4; *p* < 0.0001) and more frequent co-alterations in one or more genes in key pathways promoting cancer development/progression, which may inform therapeutic strategies.

**Abstract:**

*ARID1A* abnormalities disturb transcriptional processes regulated by chromatin remodeling and correlate with immunotherapy responsiveness. We report the first blood-based cell-free DNA (cfDNA) next-generation sequencing (NGS) *ARID1A* analysis. From November 2016 through August 2019, 71,301 patients with advanced solid tumors underwent clinical blood-derived cfDNA testing. Of these patients, 62,851 (88%) had ≥1 cfDNA alteration, and 3137 (of the 62,851) (5%) had ≥1 deleterious *ARID1A* alteration (a frequency similar to the ~6% generally reported in tissue NGS), suggesting this non-invasive test’s value in interrogating *ARID1A*. *ARID1A* cfDNA alterations were most frequent in endometrial cancer, 21.3% of patients; bladder cancer, 12.9%; gastric cancer, 11%; cholangiocarcinoma, 10.9%; and hepatocellular carcinoma, 10.6%. Blood samples with a functional *ARID1A* abnormality had more alterations/sample (median, 6 versus 4; *p* < 0.0001) and more frequent co-alterations in ≥1 gene in key oncogenic pathways: signal transduction, RAS/RAF/MAPK, PI3K/Akt/mTor, and the cell cycle. Taken together, our data suggest that liquid (blood) biopsies identify *ARID1A* alterations at a frequency similar to that found in primary tumor material. Furthermore, co-alterations in key pathways, some of which are pharmacologically tractable, occurred more frequently in samples with functional (deleterious) *ARID1A* alterations than in those without such aberrations, which may inform therapeutic strategies.

## 1. Introduction

The AT-rich interaction domain 1A (ARID1A) protein is the DNA-binding subunit in SWItch/Sucrose Non-Fermentable (SWI/SNF) chromatin remodeling complexes [1]. Alterations in genes encoding subunits of SWI/SNF complexes are of significant importance in cancer, being discerned in ~20% of human malignancies [2], with *ARID1A* loss-of-function alterations found in ~6% of solid tumor tissue samples [3].

The SWI/SNF chromatin remodeling complex controls transcription by way of an enzyme-assisted process that enables interaction with DNA by remodeling nucleosome conformation. This transcriptional regulation, combined with a high frequency of loss-of-function mutations in human cancers, points to a fundamental role of involved genes as tumor suppressors [1,4]. These observations have inspired dialog regarding how best to impact SWI/SNF chromatin remodeling aberrations such as *ARID1A* mutations in oncology.

Recent data indicate that *ARID1A* impacts anti-tumor immunity and may influence responses to multiple therapies. *ARID1A* alterations interact with the mismatch repair protein MSH2 and, therefore, attenuate mismatch repair [5]. Furthermore, *ARID1A* alterations predict better outcomes after immune checkpoint blockade in pan-cancer analysis, independent of microsatellite instability or tumor mutational burden [6]. In the Phase 3 MYSTIC study, *ARID1A* alterations predicted better outcomes of anti-PD-(L)1 monotherapy [7]. *ARID1A* has also been associated with impaired DNA damage repair, and, in colorectal cancer, *ARID1A* was enriched in patients who developed resistance to an anti-EGFR monoclonal antibody, suggesting it may play a role in resistance in this setting [8,9].

Most studies of *ARID1A* gene alterations have been conducted using tissue-based sequencing. Liquid biopsies that interrogate cell-free circulating DNA (cfDNA) via next-generation sequencing (NGS) are a non-invasive alternative to tissue biopsies. Liquid biopsy is currently used clinically for therapeutic guidance, especially when tissue testing is challenging [10,11,12,13,14]. Herein, we explore, for the first time, the blood-derived cfDNA landscape of *ARID1A* alterations, a gene gaining increasing attention for therapeutic targeting in cancer, in a pan-cancer cohort.

## 2. Materials and Methods

### 2.1. Patient Material

We queried a deidentified database containing results from consecutive patients who underwent clinical testing with a CLIA-accredited, College of American Pathologists-approved, New York State Department of Health-approved cfDNA assay (Guardant360^®^, Guardant Health, Redwood City, CA, USA). Patients with any solid tumor cancer type (as reported by the ordering physician on the test order form) tested between November 2016 and August 2019 were included in this analysis. All patients had advanced disease (stage IIIB or higher), as reported by the ordering physician. This research was conducted in accordance with ethics standards and institutional review board approval, which waived the need for informed consent to analyze deidentified data (Advarra IRB Pro00034566/CR00218935).

### 2.2. Liquid Biopsy cfDNA NGS Assay

Guardant360 is a well-validated targeted NGS cfDNA assay, with blood collection, cfDNA extraction, and sequencing procedures previously described [15,16]. Briefly, extracted cfDNA was subjected to paired-end NGS on an Illumina NextSeq500 and/or HiSeq 2500 (Illumina, Inc., San Diego, CA, USA, average read depth 15,000×) following the generation of sequencing libraries using non-random oligonucleotide adapters and hybrid capture enrichment (IDT, Inc. and Aligent Technologies, Inc.). Sequencing reads were mapped to the hg19/GRCh37 human reference sequence and were evaluated for single nucleotide variants (SNVs) in 73–74 clinically relevant cancer genes (the assay evolved from a 73-gene to a 74-gene panel over the course of the study period), as well as small insertions/deletions (indels), copy number amplifications (CNAs), and gene rearrangements/fusions in a subset of genes. The reportable ranges for SNVs, indels, fusions, and CNAs are ≥0.04%, ≥0.02%, ≥0.04%, and ≥2.12 copies, respectively, with >99.9999% per-position analytic specificity [16]. Microsatellite instability-high (MSI-H) status was available for a subset of patients upon validation of its inclusion in the assay [17].

As loss-of-function *ARID1A* alterations are considered pathogenic, only nonsense, frameshift, and canonical splice site alterations in *ARID1A* were classified as functional (implying a (deleterious) impact on function). Samples without a functional (deleterious) *ARID1A* variant were designated as wild-type *ARID1A.* Only samples with ≥1 cfDNA alteration reported were included in frequency calculations. The median number of alterations per sample was calculated using the total number of reported alterations per sample (including variants of uncertain significance and synonymous variants). Clonality was calculated using the following ratio: *ARID1A* loss-of-function alteration/maximum somatic allele fraction detected in the sample, with alterations having a ratio ≥ 0.5 defined as clonal.

### 2.3. Statistical Analysis

The frequencies of alterations in specific pathways by patient were compared using Fisher’s exact test, with variants of uncertain significance and synonymous variants excluded for the genes included in each pathway (see Table 1). Patients with MSI-H were excluded from this pathway analysis, and thus, only patients who were tested once MSI-H testing was added to the cfDNA assay were included in this analysis. SNVs, indels, splice site variants, amplifications, and fusions were assessed in the pathway analysis. A Mann–Whitney U test was performed to compare the median number of alterations per sample for samples with and without at least one functional *ARID1A* alteration. Sub-analyses were performed in the cohort of patients with NSCLC, as identified by the ordering physician on the test order form. The associations between functional *ARID1A* and several biomarkers in NSCLC were assessed, with differences in mutation frequencies compared using Fisher’s exact test. All statistical analyses were performed using GraphPad Prism version 9.1.0 for macOS (GraphPad Software, San Diego, CA, USA, www.graphpad.com).

## 3. Results

### 3.1. ARID1A Abnormalities in Blood-Derived cfDNA

**Figure 1:**
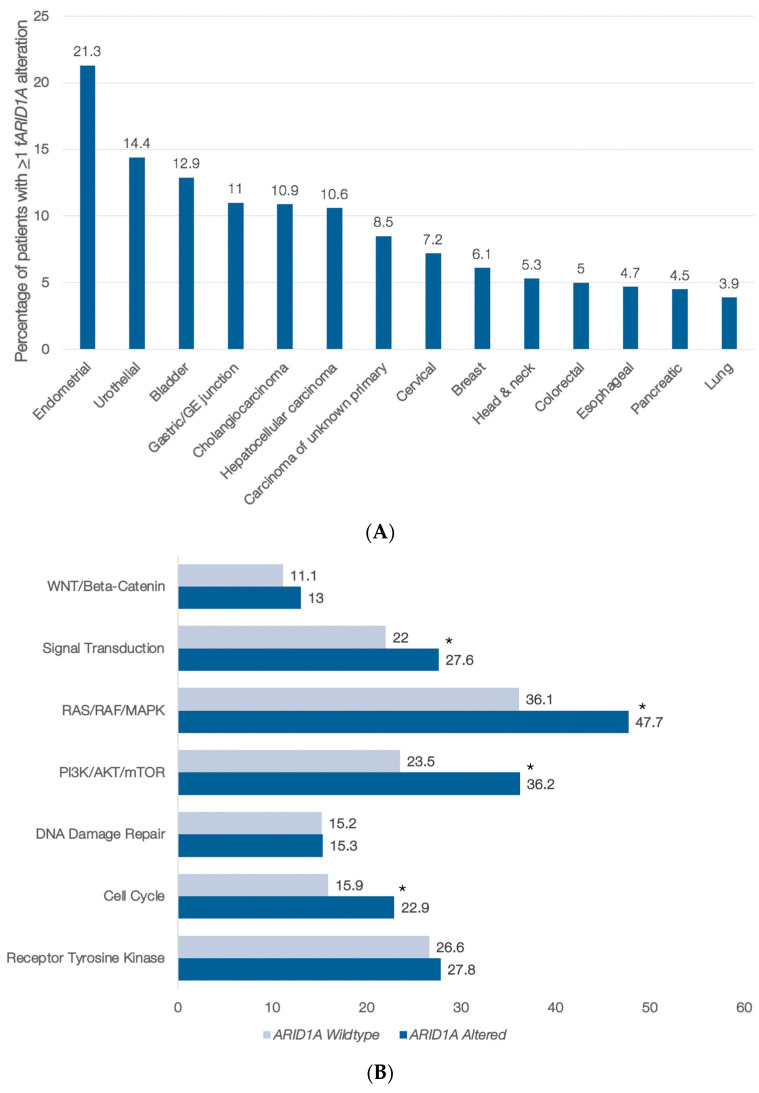
(**A**,**B**) Frequency of functional (deleterious) *ARID1A* (*fARID1A*) by cancer type and examination of co-occurring alterations by pathway. (**A**) Percentage of patients with functional *ARID1A* (*fARID1A*) cfDNA alterations by cancer type. Of 62,851 patients with ≥1 cfDNA alteration detected, 3137 (5%) had ≥1 deleterious (functional) *ARID1A* alteration (*fARID1A* alteration). Nonsense, frameshift, and canonical splice site alterations were considered functional variants. (**B**) The frequency of alterations in certain pathways was examined on a per-patient basis (i.e., counting each patient once even if they had multiple alterations in the same pathway). The denominator equates to the total number of patients with (*n* = 1088) or without (*n* = 23,935) a functional *ARID1A* alteration and at least one cfDNA alteration detected. In this pan-cancer cohort, numerous pathways were more frequently altered (statistically significant differences, *p* ≤ 0.05, marked with *, Fisher’s exact test) in patients with functional *ARID1A* genomic alterations. The genes included in each pathway are provided in Table 1. The numbers beside the bars represent percentages. For instance, the “13” beside “WNT/Beta-catenin” means that 13% of *ARID1A*-altered samples had ≥1 WNT/Beta-catenin pathway alteration. (Note: MSI-H samples were excluded from this analysis.) Abbreviations: MSI-H = microsatellite instability-high.

From November 2016 through August 2019, 71,301 patients with advanced solid tumors underwent clinical cfDNA testing; 62,851 (88%) had ≥1 cfDNA alteration; 3137 (5%) had ≥1 deleterious *ARID1A* alteration; and 50% of *ARID1A* alterations detected were clonal. The frequency of *ARID1A* alterations by cancer type is as follows (Figure 1A): endometrial cancer, 21.3% of patients; bladder cancer, 12.9%; gastric cancer, 11% (the latter includes gastric/GE junction for cfDNA); cholangiocarcinoma, 10.9%; hepatocellular carcinoma, 10.6%; carcinoma of unknown primary, 8.5%; cervical cancer, 7.2%; breast cancer, 6.1%; head and neck cancer, 5.3%; colorectal cancer, 5%; pancreatic cancer, 4.5%; and lung cancer, 3.9%. (The following were the total numbers of patients analyzed: endometrial (*N* = 517); urothelial (*N* = 146); bladder (*N* = 653); gastric/GE junction (*N* = 1764); cholangiocarcinoma (*N* = 1537); hepatocellular carcinoma (*N* = 481); carcinoma of unknown primary (*N* = 1589); cervical (*N* = 167); breast (*N* = 6880); head and neck (*N* = 582); colorectal (*N* = 5742); esophageal (*N* = 379); pancreatic (*N* = 3095); and lung (*N* = 29,378).)

### 3.2. cfDNA Genomic Alterations Co-Occurring with ARID1A Anomalies in the Pan-Cancer Setting

The number of alterations was examined as a rough surrogate of mutational burden (since many of these samples predated the availability of blood tumor mutational burden). Samples with a functional *ARID1A* alteration had a greater number of overall alterations per sample compared to those without a functional ARID1A alteration (median 6 versus 4; *p* < 0.0001), including variants of unknown significance (VUS) and synonymous variants (*ARID1A* was also included in this count) (data not shown). Tumors with versus without functional *ARID1A* alterations also more frequently harbored alterations in at least one gene in many important pathways, including signal transduction, RAS/RAF/MAPK, and the cell cycle (Figure 1B); anomalies in WNT/Beta-catenin or DNA damage repair genes were not more common in *ARID1A*-altered samples.

### 3.3. Segregation of ARID1A cfDNA Alterations with Other Oncogenic Drivers in Non-Small Cell Lung Cancer (NSCLC)

**Figure 2:**
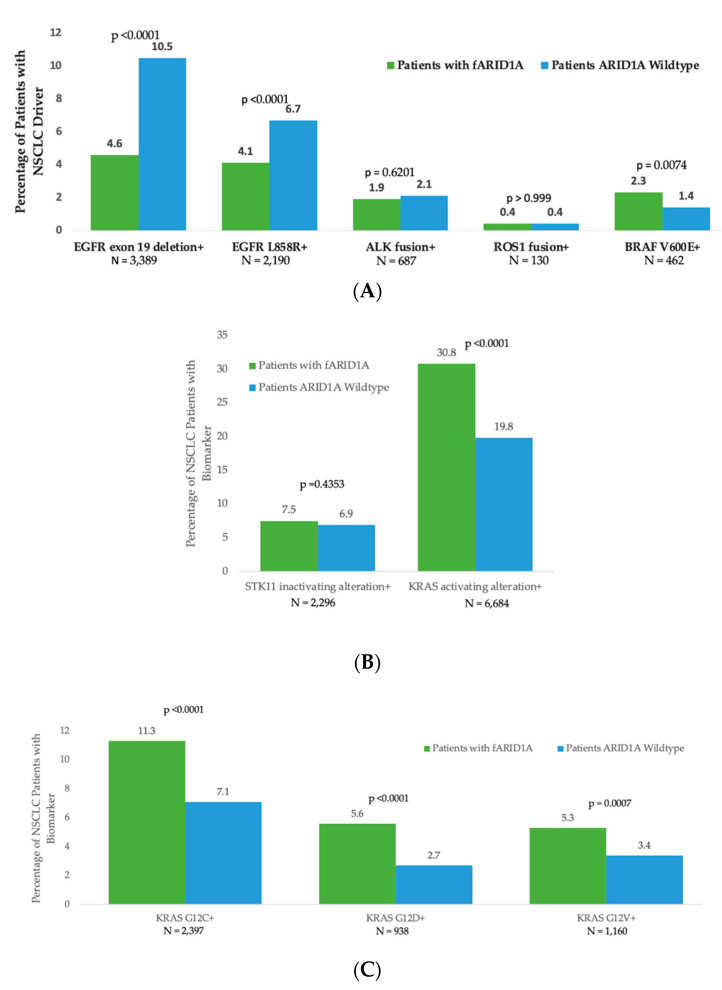
Frequency of select NSCLC biomarkers in patients with functional *ARID1A* alterations versus those without. The denominator equates to total number of NSCLC patients with (*n* = 1298) or without (*n* = 31,788) a functional *ARID1A* alteration, respectively. The number provided under each bar graph is the total number of patients in the cohort with the specified alteration. Wild-type *ARID1A* refers to samples without a functional *ARID1A* alteration. (**A**) NSCLC drivers in patients with a functional *ARID1A* alteration versus those without. For instance, 10.5% of patients without a functional *ARID1A* alteration had an *EGFR* exon 19 deletion, while only 4.6% of patients with a functional *ARID1A* alteration had such a deletion (*p* < 0.0001). (**B**) *STK11* and *KRAS* alterations (both implicated in prognosis and/or immunotherapy response) in patients without a functional *ARID1A* alteration versus those with such an alteration. (**C**)**.** Specific *KRAS* alterations in patients with functional *ARID1A* alterations versus those without.

We examined NSCLC because of the large number of targetable alterations in this tumor type [18]. In the specific subset analyses of NSCLC, certain driver alterations segregated by *ARID1A* alteration status. We specifically focused on targetable alterations and alterations implicated in resistance or sensitivity to immunotherapy [19,20]. For instance, oncogenic *EGFR* driver alterations occurred more frequently in samples without *ARID1A* versus those with functional *ARID1A* alterations, while activating *KRAS* mutations (e.g., *KRAS G12C*) were seen more frequently in samples bearing functional *ARID1A* alterations versus those without (Figure 2).

## 4. Discussion

ARID1A has a critical role in controlling gene expression that promotes carcinogenesis. Indeed, ARID1A participates in immune responsiveness to neoplastic transformation, control of PI3K/AKT/mTOR signaling, EZH2 methyltransferase activity, steroid receptor modulation, DNA damage, and modulation of KRAS and p53 signaling [12]. Many agents may theoretically be of benefit in *ARID1A*-altered malignancies: immune checkpoint blockade and inhibitors of EZH2, mTOR, histone deacetylases, PARP, and/or ATR. *ARID1A* alterations may induce resistance to platinum chemotherapy and estrogen receptor degraders/modulators [12,21,22]. Hence, *ARID1A* alterations are important to the malignant process and have therapeutic implications.

In our dataset of over 60,000 patients with advanced solid cancers, 5% had cfDNA *ARID1A* alterations in their blood-based liquid biopsy. These data are consistent with reported tissue sequencing, which shows *ARID1A* gene anomalies in approximately 6% of human malignancies [3,11,12]. Furthermore, in comparing previously published data on the frequency of *ARID1A* alterations detected via tissue testing and the current results for blood cfDNA, the frequency of *ARID1A* alterations by cancer type is as follows (tissue vs. cfDNA) [3,11,12] (Figure 1A, cfDNA shown): endometrial cancer, ~37% vs. 21.3%; bladder cancer, ~24% vs. 12.9%; gastric cancer, ~29% vs. 11% (the latter includes gastric/GE junction for cfDNA); cholangiocarcinoma, ~14% vs. 10.9%; hepatocellular carcinoma, ~9% vs. 10.6%; carcinoma of unknown primary, ~15% vs. 8.5%; cervical cancer, ~6% vs. 7.2%; breast cancer, ~5% vs. 6.1%; head and neck cancer, ~4.5% vs. 5.3%; colorectal cancer, ~7% vs. 5%.; pancreatic cancer, ~7% vs. 4.5%; and lung cancer, ~8% vs. 3.9%. Although *ARID1A* alterations occur in ~30–50% of ovarian clear cell carcinomas, ovarian clear cell cancers are a rare subset of ovarian cancer, and only one patient was designated with this pathologic entity in the dataset; hence, the frequency of *ARID1A* mutations in this histology was not evaluable [13]. It should also be noted that certain types of endometrial cancers have higher rates of *ARID1A* alterations: ~40% in uterine endometrioid carcinomas and 20–36% in uterine carcinosarcomas, but they are less frequent in endometrial serous carcinoma [14]. Our cohort did not have available details on specific histologic subtypes of endometrial cancer. Since the tissue frequencies of *ARID1A* alterations are derived from the literature, it is not clear why there are some differences in *ARID1A* alteration frequencies between tissue DNA and blood cfDNA (even if the overall frequency is similar). While certain tumor types did have higher rates of *ARID1A* alterations in the tissue than we found in the blood, others had lower frequencies: breast, ~5% vs. 6.1%; head and neck, ~4.5% vs. 5.3% (tissue versus blood). It is also plausible that tissue has been analyzed for a wider array of rare cancers that have fewer *ARID1A* alterations and that that diluted overall tissue numbers. Furthermore, certain tumor types may shed less tumor DNA into the circulation.

Blood samples with a functional (deleterious) *ARID1A* alteration had a significantly greater number of overall alterations compared to those without a functional *ARID1A* alteration (median 6 versus 4; *p* < 0.0001). Blood biopsies with versus without functional *ARID1A* alterations also more frequently harbored alterations in ≥1 gene in many important cancer-related pathways, such as signal transduction, RAS/RAF/MAPK, PI3K/Akt/mTor, and the cell cycle (Figure 1B). Importantly, some of these pathways, such as the RAS/RAF/MAPK and PI3K/Akt/mTor pathways, may be targetable by existing drugs approved or in clinical trials. The tissue gene co-alterations occurring with *ARID1A* alterations have not been extensively studied, but co-alterations in PI3K/Akt/mTor pathway genes have been noted [12]. Co-occurring pathway abnormalities may be important when planning therapy.

We did not include tumor mutational burden in the analysis because many of the samples predated the inclusion of tumor mutational burden as a blood-based analysis. In addition, we have previously shown, in multivariate analysis, that *ARID1A* alterations predicted longer PFS after checkpoint blockade, and this result was independent of microsatellite instability or mutational burden [6].

A subgroup analysis of NSCLC was also performed because of the many crucial molecular abnormalities identified in this malignancy. Specific driver alterations segregated by *ARID1A* status. As an example, oncogenic *EGFR* driver alterations, which are associated with low efficacy of immune checkpoint therapy, occurred more frequently in samples without versus those with functional (deleterious) *ARID1A* abnormalities, while activating *KRAS* mutations (e.g., *KRAS G12C*, previously considered undruggable but now therapeutically actionable with the FDA-approved sotorasib) were seen more often in blood samples harboring functional *ARID1A* (deleterious) alterations versus those without these aberrations. These findings may be notable given recent data suggesting that *KRAS* mutations may be a positive predictor of immune checkpoint response in NSCLC [19,20]. Additional research is needed to determine whether similar patterns of *ARID1A* co-alterations are seen in tumor tissue.

## 5. Conclusions

The ARID1A protein binds specific motifs of DNA as part of an SWI/SNF complex and contributes to the remarkable specificity of these chromatin remodeling complexes. These complexes exploit ATP to alter nucleosome architecture in order to hide or expose regions of DNA as part of the process mediating transcriptional regulation [12]. Through SWI/SNF complexes’ participation in the regulation of gene expression, ARID1A impacts many important cellular pathways and processes in healthy cells, and altered ARID1A plays a crucial role in the generation and propagation of the cancer state [12]. Hence, we studied ARID1A alterations in a large dataset of patients with cancer.

Taken together, our data from a validated cfDNA assay [16,17] suggest that liquid biopsies performed using this assay detect *ARID1A* alterations at frequencies similar to those found in tissue. Furthermore, at a patient level, certain co-alterations occur more frequently in samples with functional (deleterious) *ARID1A* alterations than those without such aberrations, most prominently activation via mutation of the MAPK pathway and PI3k/Akt/mTor signals. Recent studies and ongoing research suggest ARID1A may be targetable via immune checkpoint inhibitors and/or several other agents, such as PARP, EZH2, HDAC, and ATR inhibitors [12,21,22]. The impact of co-activated pathways on these strategies needs to be evaluated [23,24].

## Figures and Tables

**Table 1:** Genes used in pathway analysis (see also Figure 1B).

Pathway	Genes Included
**Receptor Tyrosine Kinase**	***EGFR*, *FGFR1*, *FGFR2*, *KIT*, *MET*, *MPL*, and *PDGFRA***
**Cell Cycle**	***CCNE1*, *CDH1*, *CDK4*, *CDK6*, *CDKN2A*, *FBXW7*, and *RB1***
**DNA Damage**	***ATM*, *BRCA1*, *BRCA2*, *CCND1*, and *MLH1***
**PI3K/AKT/mTOR**	***AKT1*, *MTOR*, *PIK3CA*, *PTEN*, *STK11*, and *TSC1***
**RAS/RAF/MAPK**	***ARAF*, *BRAF*, *ERBB2*, *GNA11*, *HRAS*, *KRAS*, *MAP2K1*, *MAP2K2*, *MAPK1*, *MAPK3*, *NF1*, *NRAS*, *RAF1*, and *RIT1***
**Signal Transduction**	***ALK*, *AR*, *DDR*, *ESR1*, *GATA3*, *GNAS*, *GNAQ*, *MYC*, *NOTCH1*,** ***NTRK1*, *NTRK3*, *PTPN11*, *RET*, *RHOA*, *ROS1*, and *SMAD4***
WNT/β **-Catenin**	***APC* and *CTNNB1***

## Data Availability

The dataset generated and/or analyzed for the current study are not publicly available, as they are derived from commercial testing. This data may be made available under a fully executed data use agreement.
